# Multilateration-based needle tracking with 3D ultrasound imaging for guiding minimally invasive procedures

**DOI:** 10.1088/1361-6560/ae5754

**Published:** 2026-04-07

**Authors:** Weidong Liang, Christian Baker, Javad Rostami, Simeon West, Athanasios Diamantopoulos, Sebastien Ourselin, Laura Peralta, Wenfeng Xia

**Affiliations:** 1 School of Biomedical Engineering & Imaging Sciences, King’s College London, London, United Kingdom; 2 Department of Anaesthesia, University College London Hospital, London, United Kingdom; 3 Department of Interventional Radiology, Guy’s & St Thomas’ NHS Foundation Trust, London, United Kingdom

**Keywords:** chirp excitation, fiber-optic hydrophone, multilateration, needle tracking, sparse array, 3D ultrasound imaging

## Abstract

*Objective.* Ultrasound (US) is commonly used to guide minimally invasive procedures; however, its effectiveness is often limited by poor needle-tip visibility. This work presents a 3D US imaging and needle-tip tracking system based on US multilateration, toward real-time, safe and efficient guidance. *Approach.* The proposed system integrates a fiber-optic hydrophone within a medical needle to receive US transmissions from a sparse 2D matrix array probe used for 3D imaging. Chirp excitation was employed to improve the signal-to-noise ratio and enable robust time-of-arrival estimation. A multilateration algorithm was employed for needle-tip localization relative to the US probe, requiring fewer US elements and achieving substantially faster performance than existing methods. *Main results.* The system achieved spatial tracking accuracy of $1.27 \pm 0.65$ mm using only 13 active elements, representing an order-of-magnitude increase in tracking speed from 3.77 to 45.45 Hz compared to a conventional delay-and-sum-based tracking algorithm with 256 elements. The translational potential of the system was further demonstrated with *ex vivo* chicken tissue, and a clinically realistic femoral nerve block phantom. *Significance.* These results demonstrate the feasibility of a multilateration-based system that combines 3D anatomical visualization with needle tracking. With further improvements towards real-time operation and validation under clinically relevant conditions, this approach could improve the precision and safety of US-guided interventions.

## 1. Introduction

US imaging is widely used to guide minimally invasive procedures such as nerve blocks, tumor biopsies and fetal surgeries due to its real-time imaging capability, absence of ionizing radiation, portability, and cost-effectiveness (Kumar and Brien 2004, Chin *et al* 2008, O’Flynn *et al* 2010, Roy-Chowdhuri *et al* 2017). However, despite these advantages, needle visibility remains a major challenge that critically affects the safety and accuracy of US-guided interventions. First, needle visibility is often poor due to specular reflection from the smooth shaft, particularly at steep insertion angles, where tip ‘dropout’ can occur, leading to uncertainty in tip location (Chin *et al* 2008). Second, needle visibility can be severely reduced in highly echogenic tissues, making it difficult to distinguish the needle from surrounding structures (Reusz *et al* 2014). As these procedures are typically guided by 2D US imaging, out-of-plane insertions can cause the intersection of the needle shaft with the imaging plane to be misinterpreted as the needle tip (Souza *et al* 2018). Inaccurate needle placement during interventional procedures can cause serious complications, including vascular injury, organ damage, nerve trauma, infection, and diagnostic failure, all of which can increase patient morbidity and may necessitate further interventions (Chin *et al* 2008, Bhatia *et al* 2010, O’Flynn *et al* 2010).

To address these challenges, several techniques have been adopted in clinical practice. For example, in nerve block procedures, echogenic needles with specialized surface coatings are commonly used to enhance acoustic scattering and improve needle visibility under US (Hebard and Hocking 2011). However, such coatings may introduce image artifacts, thereby complicating accurate tip localization, and 3D needle visualization remains challenging. Mechanical needle guides provide a fixed insertion trajectory and reduce dependence on operator skill, but they restrict free-hand maneuverability and are less favored by experienced clinicians (Sato *et al* 2024). Software-based solutions have also been explored; for example, automated needle detection algorithms applied to real-time US imaging have shown promise, but their performance remains limited by image quality and can be confounded by surrounding anatomical structures with similar linear features (Zhao *et al* 2013, Maneas *et al* 2021). Volumetric US imaging can be reconstructed from free-hand US acquisitions to support navigation (Lang *et al* 2012, Tang *et al* 2021). However, these approaches are generally not real-time, which limits their applicability for intraoperative guidance.

Active needle tracking has emerged as a key research focus in recent years, typically involving the integration of a miniature sensor within the needle to actively localize its position during interventional procedures (Ramadani *et al* 2022). One common approach employs electromagnetic (EM) trackers mounted on the needle, which estimate spatial location by detecting the gradient of the external EM field (Fenster and Downey 2000, Reusz *et al* 2014, Seitel *et al* 2024). However, such systems are often susceptible to distortion from surrounding metallic instruments, and the EM field generators are typically bulky. Piezoelectric US transducers at the needle tip enable real-time intraoperative localization (Breyer and Cikeš 1984, Mung *et al* 2011a, 2011, Guo 2015). Nevertheless, miniaturizing piezo-components for integration at the needle tip can be challenging and costly, and piezo-based transmitters usually require high-voltage excitation, posing potential safety risks. Fiber-optic US sensing offers a promising alternative for active needle tracking, owing to its cost-effectiveness, ease of miniaturization, and omnidirectional sensitivity. In 2015, Xia *et al* demonstrated a needle-tip tracking system using a fiber-optic hydrophone (FOH) embedded in a 20-gauge needle for tracking with 2D B-mode US imaging (Xia *et al* 2015). However, tracking was restricted to 2D due to the use of a 1-D array US imaging probe. Although a later system extended the method to 3D localization using a customized probe (Xia *et al* 2017), it did not provide real-time volumetric imaging of patient anatomy together with the needle trajectory.

Recently, we developed a system that integrates volumetric US imaging with 3D needle tracking, using a 256-element 2D sparse array and an FOH embedded in the needle (Rostami *et al* 2024, Liang *et al* 2026). Volumetric imaging was achieved via coherent plane-wave (PW) imaging, while the FOH sequentially recorded tracking data from 256 pulses transmitted by each of the active elements of the spiral array. Needle tip location was then estimated as the center-of-mass (CoM) of the beamformed 3D tracking image. However, the tracking frame rate was constrained by the sequential excitation of a large number of transducer elements required for accurate localization, limiting its applicability for real-time intraoperative guidance.

In this work, we address this limitation by developing a multilateration-based localization algorithm using a substantially reduced number of US elements, toward real-time 3D needle tracking. Chirp excitation with matched filtering (pulse compression) was applied to enhance the signal-to-noise ratio (SNR) and improve time-of-arrival (ToA) estimation. By reducing the number of active elements and the associated computational load, the proposed method achieves significantly faster tracking while preserving accurate localization, thereby advancing its potential for clinical translation.

## 2. Material and methods

### 2.1. System overview

A detailed description of the proposed system is provided in Liang *et al* (2026). Briefly, the system is based on a 256-channel US Open Platform (ULA-OP 256, MSD Laboratory, Department of Information Engineering, University of Florence, Italy) (Boni *et al* 2016, Mazierli *et al* 2021), which was used to drive a 2D matrix array (Vermon S.A., Tours, France) comprising $32 \times 35$ elements (1024 in total). A designated subset of 256 elements, arranged in a sparse Fermat’s spiral pattern (Ramalli *et al* 2015, Peralta *et al* 2023), was hardwired to a connector interfacing with the ULA-OP 256 to represent a sparse 2D array probe for 3D US imaging. For 3D tracking, a FOH was integrated into the cannula of a 20-gauge, 150 mm-long needle (assembled by OxDevice Ltd., Abingdon, UK) to receive US transmissions from the imaging probe. The FOH operates on a Fabry–Pérot interferometric principle, using a cavity formed by two parallel reflective surfaces. A tunable laser (SL-550, Santec, Japan; 1500–1600 nm wavelength range) interrogates the cavity through the fiber. When acoustic waves impinge on the cavity, the resulting pressure deforms it, modulating its spacing and altering the interference pattern to enable precise pressure detection (Morris *et al* 2009).

**Figure 1.**
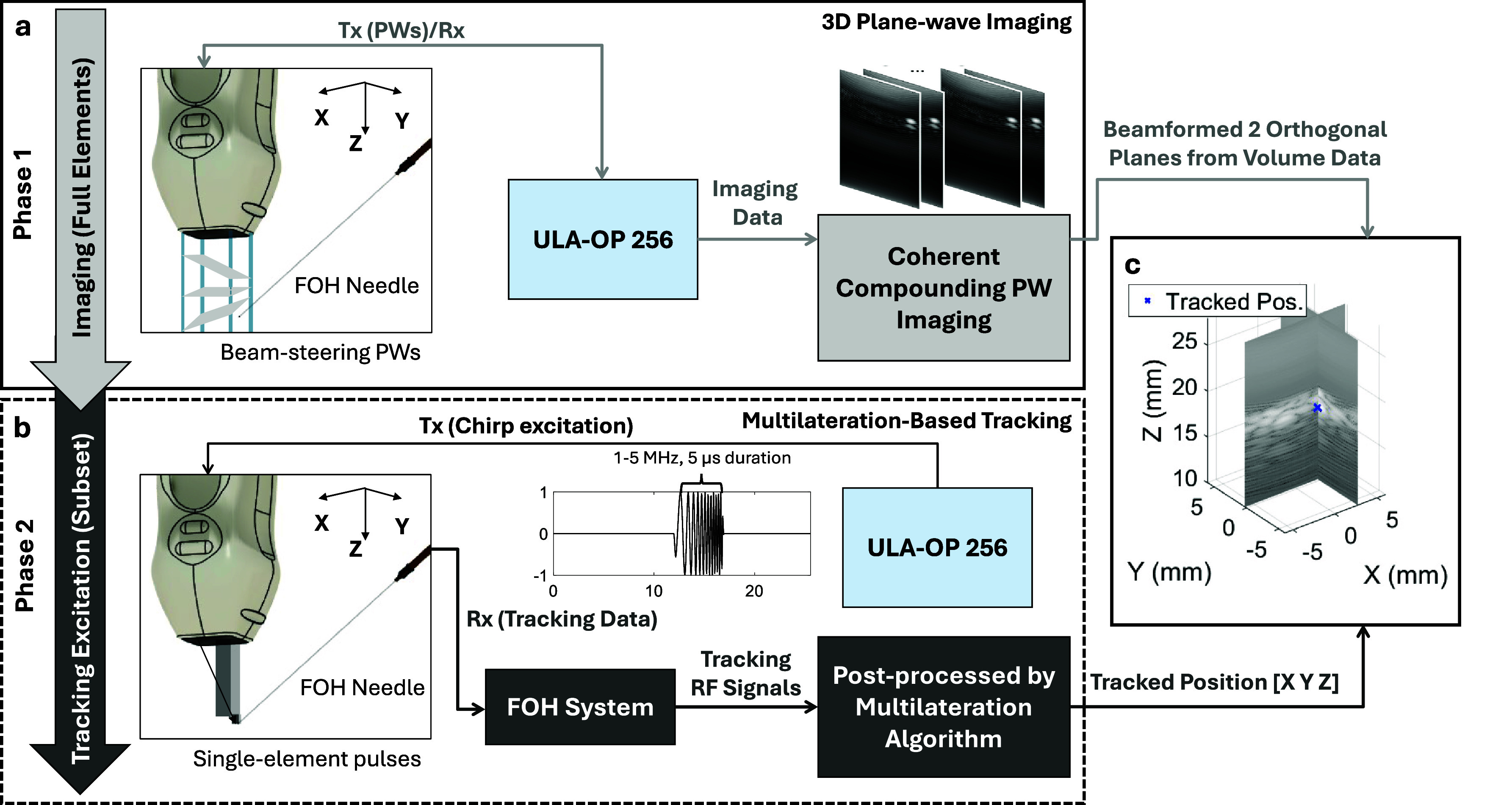
Schematic illustration of the system. Each frame of the system comprises two phases. (a) phase 1: plane wave imaging. The ULA-OP 256 drives the 2D matrix array to transmit nine beam-steered plane waves in different directions. The received US data are beamformed and coherently compounded to generate real-time 3D US images, displayed in two orthogonal planes of the field of view (FoV). (b) Phase 2: ultrasonic tracking. Individual element from a selected subset of the probe is sequentially excited using a chirp signal, and the transmitted US signals are received by the fiber-optic hydrophone (FOH). A multilateration algorithm processes the time-of-arrival data to directly estimate the 3D tip position [*X*, *Y*, *Z*]. (c) The estimated needle tip position is superimposed on the 3D US image for visualization.

During system operation, each frame consisted of two phases: imaging (Phase 1) and tracking (Phase 2), as illustrated in figure 1. In Phase 1 (figure 1(a)), the ULA-OP 256 drove all 256 active elements to transmit nine beam-steered PWs, covering steering angles from $-5\,^\circ$ to $5\,^\circ$ in 2.5$\,^\circ$ steps along both lateral and elevational directions. Four-cycle Gaussian pulses at a 3 MHz center frequency were transmitted at a pulse repetition frequency (PRF) of 1 kHz, and raw channel data were acquired at 26 MHz. The received echoes from each PW steering angle were beamformed, and beamformed US images from all PW transmissions were coherently compounded to compute the volumetric dataset. Two orthogonal B-mode slices were simultaneously displayed in real-time.

In Phase 2 (figure 1(b)), a sparsified subset of the 256 array active elements was sequentially activated to transmit chirp pulses at 1 kHz PRF. Each pulse consisted of a 5 *µ*s linear frequency sweep from 1 to 5 MHz, while the FOH embedded in the needle received the transmissions. The acquired FOH data were then processed using a multilateration-based algorithm, originally developed for GPS positioning (Torrieri 2007), to estimate the 3D needle tip coordinates $[X,Y,Z]$. The tracked position was subsequently overlaid onto the volumetric US image obtained in Phase 1 (figure 1(c), marked with an ‘×’).

### 2.2. Signal processing

**Figure 2.**
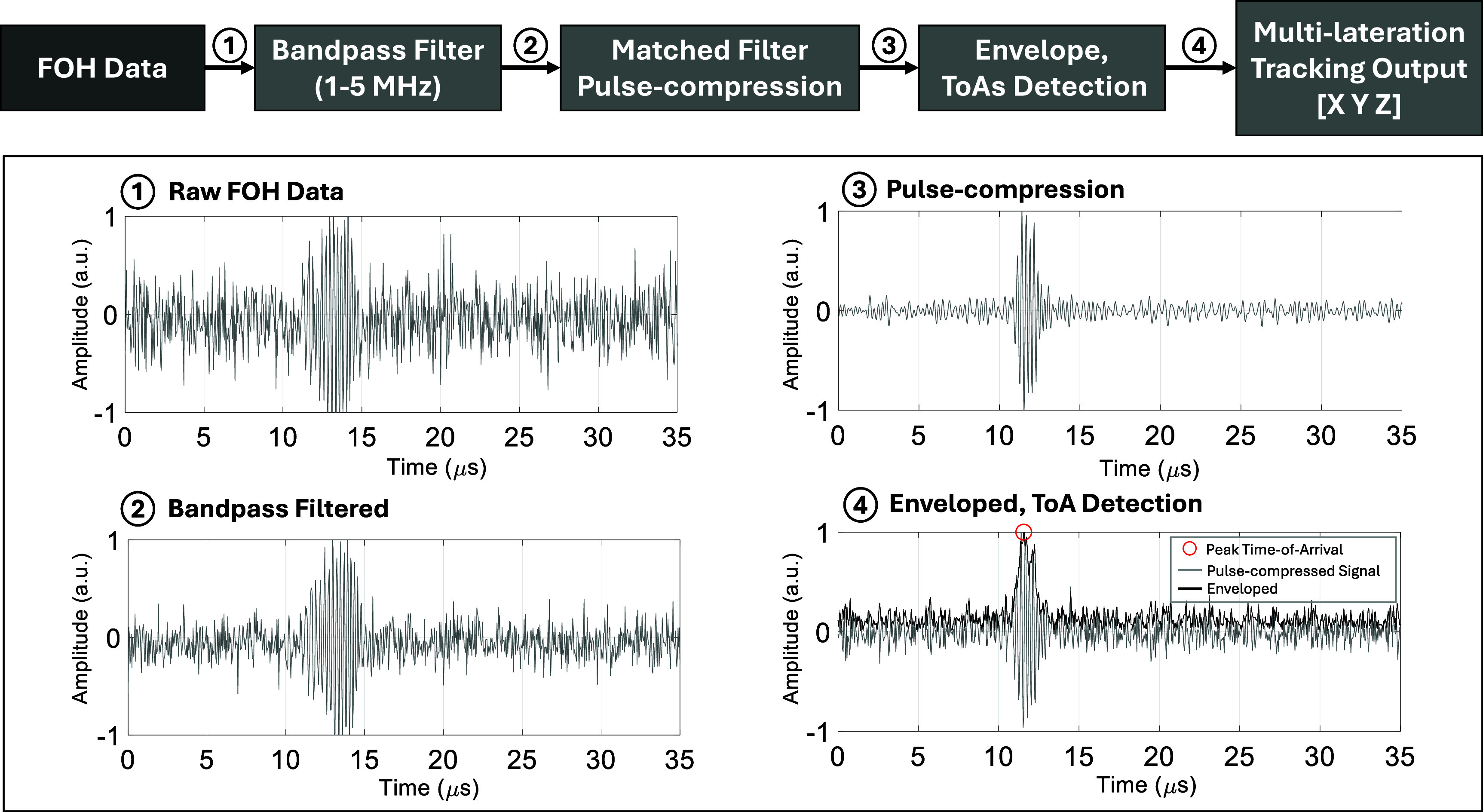
Signal processing pipeline for the multilateration-based tracking system. Chirp-excitation RF signals captured by the fiber-optic hydrophone (FOH) are bandpass filtered (1–5 MHz), pulse-compressed via matched filtering, and envelope-detected extract the time-of-arrival (ToA).

The complete tracking signal processing pipeline is illustrated in figure 2. The FOH signals were first bandpass-filtered (1–5 MHz) to suppress out-of-band noise, followed by pulse compression using a time-reversed matched filter to enhance the SNR (section 2.2.1). An envelope detector was then applied and the peak location of the resulting signal was used to estimate the ToA (section 2.2.2). These ToA estimates were subsequently used as input to the multilateration algorithm, which estimated the 3D position of the FOH relative to the probe coordinate system (section 2.2.3).

#### 2.2.1. SNR enhancement with chirp excitation

To improve the reliability of ToA measurements and enhance multilateration accuracy, it is essential to increase the SNR of the FOH data. In this study, a 5 *µ*s linear chirp excitation ranging from 1 to 5 MHz was employed. The received FOH signal was subsequently processed using a pulse-compression scheme to improve the SNR. The compressed signal $ s_{\mathrm{pc}}(t) $ was obtained by cross-correlating the received signal *r*(*t*) with the time-reversed transmitted chirp $ c(-t) $, given by:

\begin{equation*} s_{\mathrm{pc}}\left(t\right) = r\left(t\right) \ast c\left(-t\right)\end{equation*}

where $ \ast $ denotes the cross-correlation operation.

#### 2.2.2. ToA estimation

**Figure 3.**
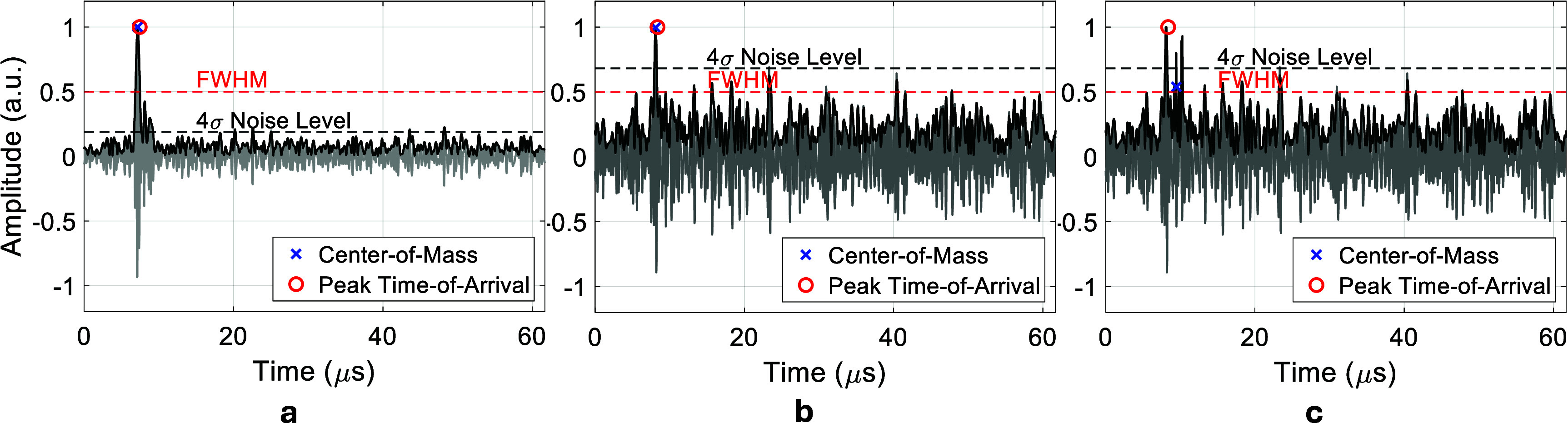
Signal exclusion criteria for accurate time-of-arrival (ToA) estimation. (a) A valid signal with a full width at half maximum (FWHM) exceeding $4\,\sigma$ and a time delay between the peak and center-of-mass (CoM) below 2 *µ*s. (b) A noisy signal with FWHM below $4\,\sigma$, considered unreliable and excluded. (c) A signal with a peak-to-CoM delay exceeding 2 *µ*s, also rejected. $\,\sigma$: baseline noise level (standard deviation).

During intraoperative conditions, multipath reflections from tissue boundaries, along with ambient noise, can corrupt the reliability of ToA estimation. To address this, a ToA estimation method (see figure 3 and algorithm 1) was developed for multilateration tracking.

**Table:** 

**Algorithm 1.** Time-of-arrival estimation.
**function** DetectReliableToAs(tracking_RF_signal)
accepted_ToAs $\gets \{\}$
**for** each *s* in tracking_RF_signal **do**
$s_{\mathrm{env}} \gets \mathrm{Envelope}(s)$
$\mathrm{PeakToA} \gets \mathrm{FindPeakTime}(s_{\mathrm{env}})$
$A_{50} \gets 0.5 \cdot \max(s_{\mathrm{env}})$ ▹ 50% peak threshold
$\mathrm{HW} \gets \mathrm{HalfMaxWidth}(s_{\mathrm{env}}, A_{50})$ ▹ Width above *A*_50_
$\mathrm{CoM} \gets \mathrm{ComputeCenterOfMass}(s_{\mathrm{env}} \unicode{x2A7E} A_{50})$
$\sigma \gets \mathrm{std}\!\left(s_{\mathrm{env}}[t < 5~\mu\mathrm{s}]\right)$
**if** $\mathrm{HW} < 4\,\sigma$ **then**
**continue** ▹ Reject noisy signal
**end if**
**if** $\left|\mathrm{CoM} - \mathrm{PeakToA}\right| > 2~\mu\mathrm{s}$ **then**
**continue** ▹ Reject inconsistent signal
**end if**
accepted_ToAs.append($\mathrm{PeakToA}$)
**end for**
**return** accepted_ToAs
**end function**

The process consisted of five steps:

1. **Peak detection:** Identify the ToA peak from the enveloped RF signal.
2. **full width at half maximum (FWHM) calculation:** Measure the FWHM of the peak.
3. **Signal CoM:** Compute the CoM for the signal content above the FWHM (-6 dB) threshold.
4. **Noise estimation:** Estimate the baseline noise *σ* from the initial 5 *µ*s where no signal is expected.
5. **Filtering criteria:**
  • Discard signals with FWHM $ < 4\,\sigma$.
  • Discard signals with a peak-to-CoM delay $ > 2$ *µ*s.

Only signals passing these steps were used in multilateration, improving the accuracy and robustness of the tracking output.

#### 2.2.3. Multilateration needle tracking

This work employed a multilateration-based method to directly estimate and track the spatial position of the needle-integrated FOH, in contrast to our previous approach (Liang *et al* 2026), which relied on delay-and-sum (DAS) beamforming. In the DAS framework, FOH signals were reconstructed into a volumetric image by coherently summing time-aligned echoes across the array, and the needle-tip position was subsequently derived from the center of mass of the reconstructed intensity distribution. While effective, this strategy is computationally demanding, as it requires full 3D image reconstruction before localization. Moreover, sequential excitation of all 256 elements in the sparse array further increases acquisition time, thereby limiting its applicability for intraoperative guidance. By contrast, multilateration provides a more direct and efficient solution. In this method, ToA measurements from sequential single-element transmissions from a subset of the sparse array were used to calculate the propagation distances between the FOH and each element in a sparsified tracking array with known spatial coordinates in the imaging reference frame. Each estimated distance defined the radius of a sphere centered at the corresponding tracking element, and the intersection of these spheres provided an estimate of the FOH tip position. A minimum of three active elements is required to uniquely determine the spatial location in $[X,Y,Z]$ based on their corresponding ToA measurements. Incorporating additional elements with known ToAs yields an overdetermined least-squares formulation, thereby enhancing robustness against measurement noise and timing uncertainties. This principle is analogous to GPS trilateration, where satellite signals are fused to determine receiver position in three dimensions.

Let the positions of *n* known transmitters be defined as:

\begin{equation*} \mathbf{s}_i = \begin{bmatrix} x_i \\ y_i \\ z_i \end{bmatrix}, \quad i = 1, 2, \dots, n.\end{equation*}

Let the unknown 3D position of the FOH be:

\begin{equation*} \mathbf{p} = \begin{bmatrix} x \\ y \\ z \end{bmatrix}.\end{equation*}

Assuming a constant speed of sound *c*, and ToA measurements *t_i_* from each transmitter, the corresponding distance is:

\begin{equation*} d_i = c \cdot t_i.\end{equation*}

Each ToA measurement defines a nonlinear constraint:

\begin{equation*} \| \mathbf{p} - \mathbf{s}_i \| = d_i = c \cdot t_i.\end{equation*}

The residual for each transmitter is:

\begin{equation*} r_i = \| \mathbf{p} - \mathbf{s}_i \| - d_i.\end{equation*}

The position **p** was estimated by minimizing the sum of squared residuals:

\begin{equation*} \min_{\mathbf{p}} \sum_{i = 1}^{n} \left( \| \mathbf{p} - \mathbf{s}_i \| - c \cdot t_i \right)^2.\end{equation*}

A Gauss–Newton iterative least-squares method was then applied to obtain the best estimate of the unknown position **p** (Baker *et al* 2024).

### 2.3. Multilateration tracking with sparsified subsets

**Figure 4.**
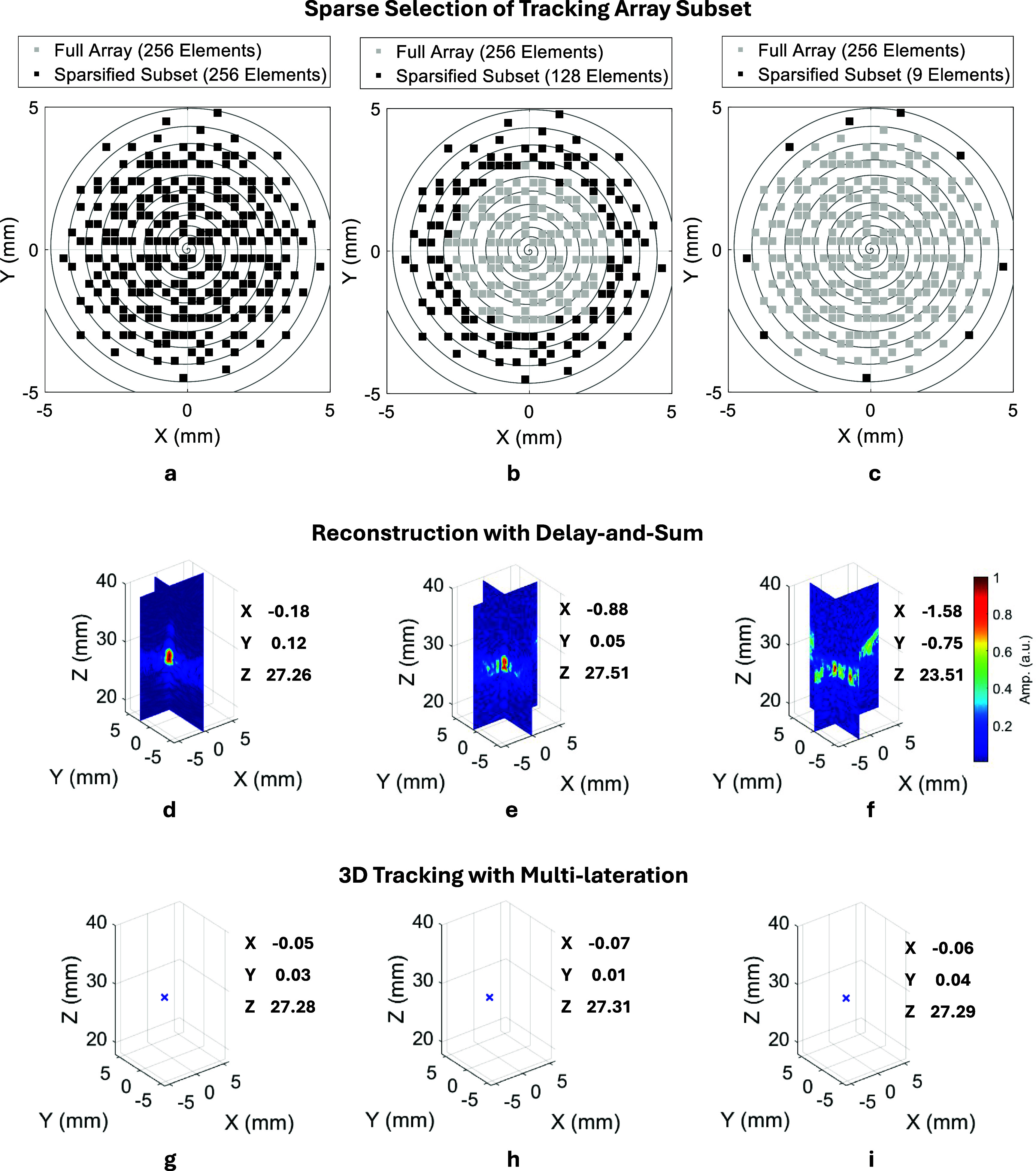
Tracking performance using sparsified subsets of matrix array elements. (a)–(c): Layout of the spiral ultrasound array, with the elements activated for tracking highlighted in black for different sparsification conditions. (d)–(f): Tracking subset sparsification strategy from the full 256 elements to the outermost 9 elements, following the Fermat’s spiral layout-from inner to outer layers. (d)–(f): Delay-and-Sum (DAS)-reconstructed 3D tracking images of the needle tip using the same water tank dataset, corresponding to subsets in (a)–(c). Reduced element count introduces artefacts and degrades image contrast, affecting tracking precision. (g)–(i): Tracked positions derived using the proposed multilateration method from the same dataset.

To enhance the system’s clinical translation potential, improving the data acquisition rate is essential for increasing the overall imaging-tracking frame rate. To this end, the effect of tracking array sparsification on localization accuracy was investigated. A sparsification strategy was applied during post-processing to the original 256-element dataset by progressively discarding signals from selected channels, following a Fermat’s spiral pattern from inner to outer elements (figure 4, top row). The number of active channels was reduced from 256 to 3, the minimum required for 3D multilateration. While the resulting frame rate for data acquisition under the proposed sparsification scheme was estimated as

\begin{equation*} f_\mathrm{acq} = \frac{\mathrm{PRF}}{N_\mathrm{img} + N_\mathrm{trk}},\end{equation*}

where $\mathrm{PRF} = 1000$ Hz is the PRF, $N_\mathrm{img}$ is the number of imaging transmissions (9 in this study), and $N_\mathrm{trk}$ is the number of tracking transmissions, equal to the number of active elements in the selected subset.

FOH data were collected with the needle tip immersed in water (see tracking accuracy measurement in section 2.4 below). The tracking accuracy and frame rate of two methods were compared using varying numbers of active elements: (1) DAS beamforming to reconstruct 3D needle-tip images (Liang *et al* 2026), and (2) the proposed multilateration approach for direct estimation of spatial coordinates $[X, Y, Z]$.

### 2.4. Performance evaluation

To evaluate the tracking accuracy of the proposed multilateration method, experiments were conducted in a water tank setup. As described in Liang *et al* (2026), the FOH-integrated needle was mounted on a motorized translational stage, with the imaging probe positioned downward and partially submerged. After registering the motion axes to the probe’s coordinate system, the needle was translated along a predefined trajectory from 10 to 40 mm depth in 10 mm increments. At each depth, tracking data were acquired at 9 lateral/elevational positions on a 2 mm grid. At all testing positions, tracking signals from all 256 array elements were acquired to form a complete dataset. During post-processing, sparsified subsets of these channels were systematically selected by progressively disabling elements from the inner layers of the array towards the outer layers, following the Fermat’s spiral geometry of the transducer (see figures 4(a)–(c)). This strategy enabled a controlled evaluation of multilateration-based tracking performance using different numbers and spatial distributions of active elements, while keeping the experimental acquisition identical across all conditions.

To assess the SNR enhancement achieved through chirp excitation and pulse-compression scheme, needle insertions were conducted in *ex vivo* chicken breast tissue (see figure 6(g). To account for variability in tissue properties and insertion conditions, tracking data were collected from 3 independent needle insertion attempts, with 3 repeated measurements acquired at each testing location. Two excitation modes were implemented for controlled comparison. In the first mode, a four-cycle Gaussian tone-burst pulse at 3 MHz was transmitted. In the second mode, a 5 *µ*s linear chirp sweeping 1–5 MHz was used, followed by pulse compression with a time-reversal matched filter to enhance the SNR.

Tracking performance was further evaluated using a femoral nerve block phantom (VALKYRIE Simulators, USA) to demonstrate the system’s ability to perform 3D tracking and volumetric imaging in a clinically realistic setting (figure 8(c)). The FOH needle was manually inserted under US guidance at an angle of approximately 45$\,^\circ$ to the phantom surface, simulating a clinically relevant oblique approach. Three repeated insertion attempts were performed. During each attempt, the needle was advanced in small increments, and three spatially distinct locations along the shaft trajectory were randomly selected for signal acquisition. At each site, both tone-burst and chirped excitations were transmitted sequentially, with corresponding FOH tracking signals recorded. Volumetric US imaging frames were also acquired using the imaging probe. All data were stored for subsequent post-processing, where tracked positions were overlaid onto the corresponding volumetric images for comparison.

## 3. Results

### 3.1. Accuracy assessment

**Figure 5.**
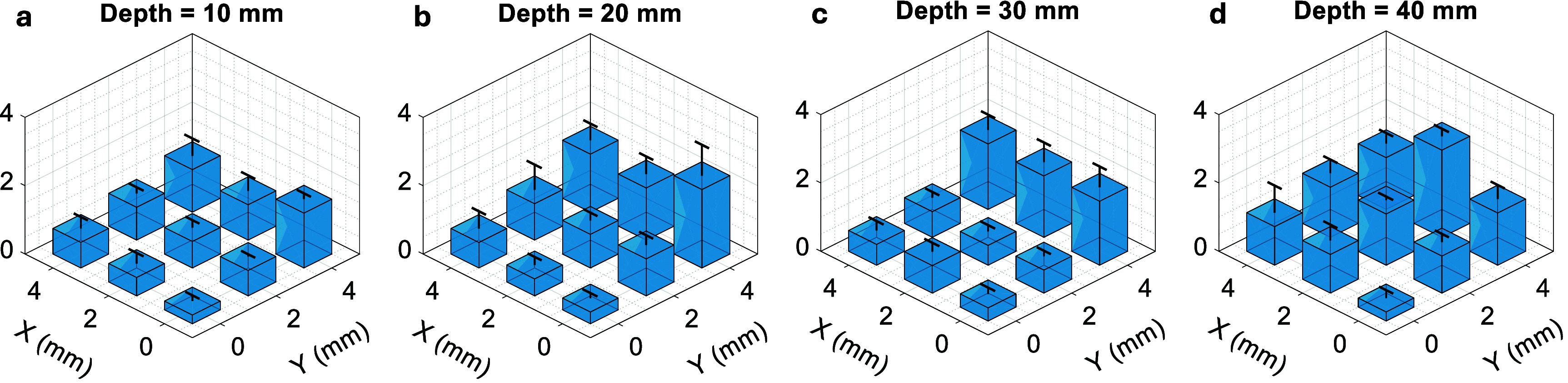
Tracking accuracy with only 13 active US elements measured at multiple spatial locations in a water bath. The needle with integrated fiber-optic hydrophone was positioned using a motorized translational stage at depths from 10 to 40 mm (a) and (b). Error bars indicate the standard deviation from three repeated measurements at each location. More detailed quantitative results are summarized in table 1.

Figure 5 shows the tracking accuracy measured at various spatial locations using only 13 active US elements (corresponding quantitative results are presented in table 1), with the target position offset laterally from the probe center toward the edges and across different imaging depths. At the central position (0, 0), the mean tracking accuracy, averaged across all tested depths (10–40 mm), was $0.31 \pm 0.09$ mm. In contrast, at the peripheral position (4, 4), the corresponding mean error increased to $1.74 \pm 0.82$ mm, indicating a systematic degradation in tracking accuracy toward the edges of the measurement field of view (FoV). Depth-dependent analysis showed mean errors of $1.01 \pm 0.31$ mm, $1.21 \pm 0.51$ mm, $1.27 \pm 0.60$ mm, and $1.36 \pm 0.43$ mm at 10 mm, 20 mm, 30 mm, and 40 mm depths, respectively. This gradual increase in error with both lateral offset and depth is consistent with the effects of limited probe aperture, reduced angular coverage for off-axis targets, and acoustic attenuation over longer propagation paths. Overall, the tracking accuracy, averaged across all the spatial positions, was $1.27 \pm 0.65$ mm, supporting the feasibility of the approach for precise needle-tip localization.

**Table 1.** Estimated needle-tip tracking accuracy in water with only 13 active US elements, reported as mean ± standard deviation (mm), at different lateral positions and depths. Rows correspond to lateral offsets in the *X* direction and columns correspond to offsets in the *Y* direction.

		Y (mm)		
		0	2	4
X (mm)	**Depth = 10 mm**			
	0	$0.24 \pm 0.08$	$0.81 \pm 0.10$	$1.18 \pm 0.16$
	2	$0.58 \pm 0.10$	$1.12 \pm 0.19$	$0.92 \pm 0.11$
	4	$0.71 \pm 0.12$	$1.29 \pm 0.18$	$1.55 \pm 0.17$
	**Depth = 20 mm**			
	0	$0.38 \pm 0.10$	$1.02 \pm 0.12$	$1.82 \pm 0.28$
	2	$0.61 \pm 0.11$	$1.39 \pm 0.21$	$1.56 \pm 0.20$
	4	$0.83 \pm 0.12$	$2.08 \pm 0.30$	$1.94 \pm 0.31$
	**Depth = 30 mm**			
	0	$0.39 \pm 0.10$	$0.74 \pm 0.12$	$1.48 \pm 0.21$
	2	$0.64 \pm 0.12$	$1.93 \pm 0.29$	$1.63 \pm 0.22$
	4	$0.72 \pm 0.13$	$2.18 \pm 0.33$	$1.84 \pm 0.28$
	**Depth = 40 mm**			
	0	$0.33 \pm 0.11$	$1.17 \pm 0.20$	$1.52 \pm 0.24$
	2	$0.83 \pm 0.15$	$2.46 \pm 0.39$	$2.32 \pm 0.31$
	4	$1.08 \pm 0.21$	$2.71 \pm 0.41$	$1.62 \pm 0.23$

### 3.2. Frame-rate enhancement

To compare the tracking performance under array sparsification, two tracking algorithms were evaluated: (1) DAS beamforming and (2) the proposed multilateration approach. For each sparsified subset, tracking accuracy was quantified by comparing the estimated position with the ground truth from the translational stage. Using DAS, the tracking accuracy degraded rapidly from 1.24 mm, to 1.42 mm and 7.43 mm, as the number of active elements decreased from 256 to 128 and 9, respectively (figures 4(d)–(f)). In contrast, the multilateration method maintained reliable; the tracking accuracy only varied from 1.26 mm to 1.52 mm with the channel count reduced significantly from 256 to 9 (figures 4(g)–(i)).

**Figure 6.**
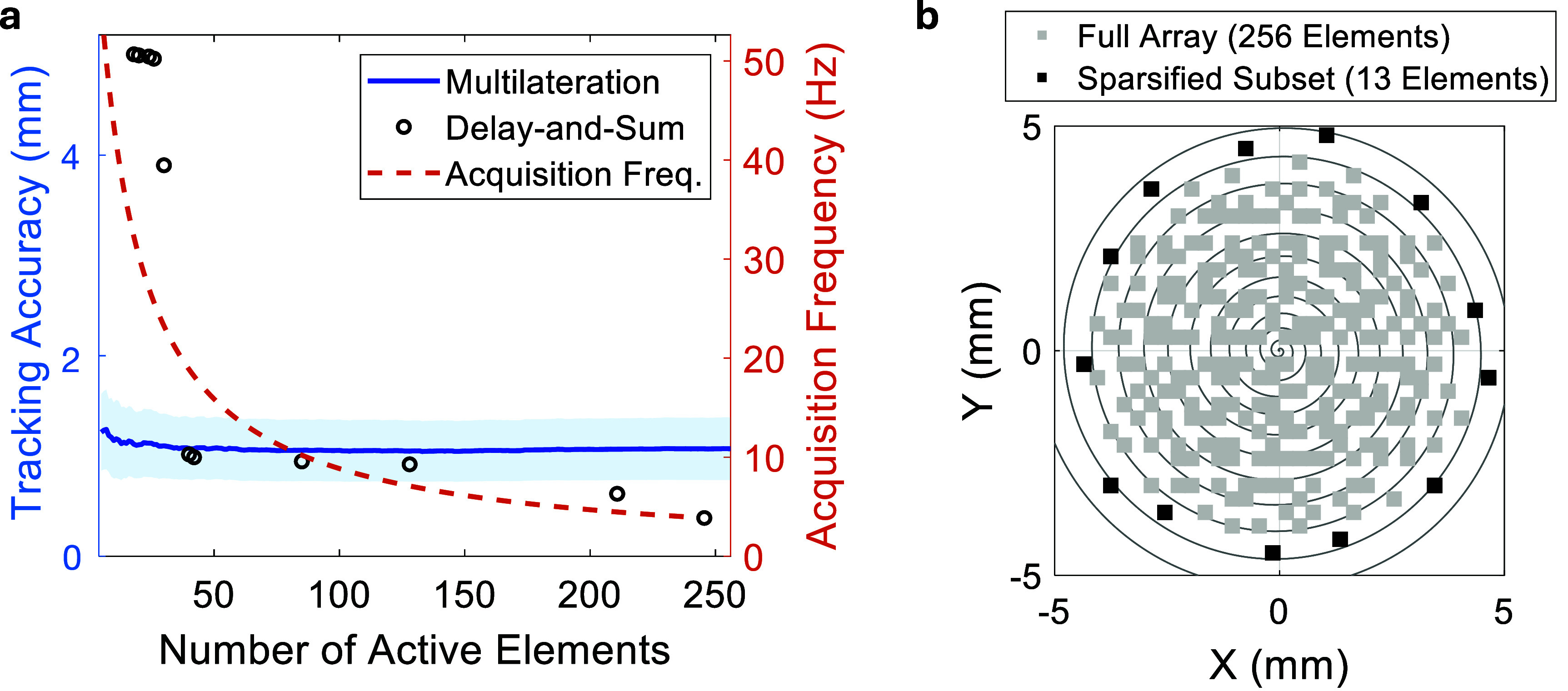
Comparative tracking accuracy of DAS and multilateration methods under progressive sparsification of tracking elements. (a) Mean localization (relative to translational stage ground truth) as the number of active elements is reduced from 256 to the minimum 3, following the Fermat’s spiral pattern layout in figure 4(top row). The corresponding acquisition frequency is also plotted as a function of active element count. (b) Example sparsification layout retaining the 13 outermost elements.

Using data collected in the water tank, figure 6(a) compares the tracking accuracy and acquisition frame rate of both methods under identical sparsification schemes. With only 13 active elements (outermost Fermat spiral layer, figure 6(b), the system achieved a mean tracking accuracy of $1.27 \pm 0.65$ mm, while the theoretical acquisition frame rate increased from 3.77 Hz (full 256 elements) to 45.45 Hz. These findings demonstrate the robustness of multilateration for low-channel-count, high-speed tracking and highlight its promise for real-time clinical translation.

### 3.3. Ex vivo tissue

**Figure 7.**
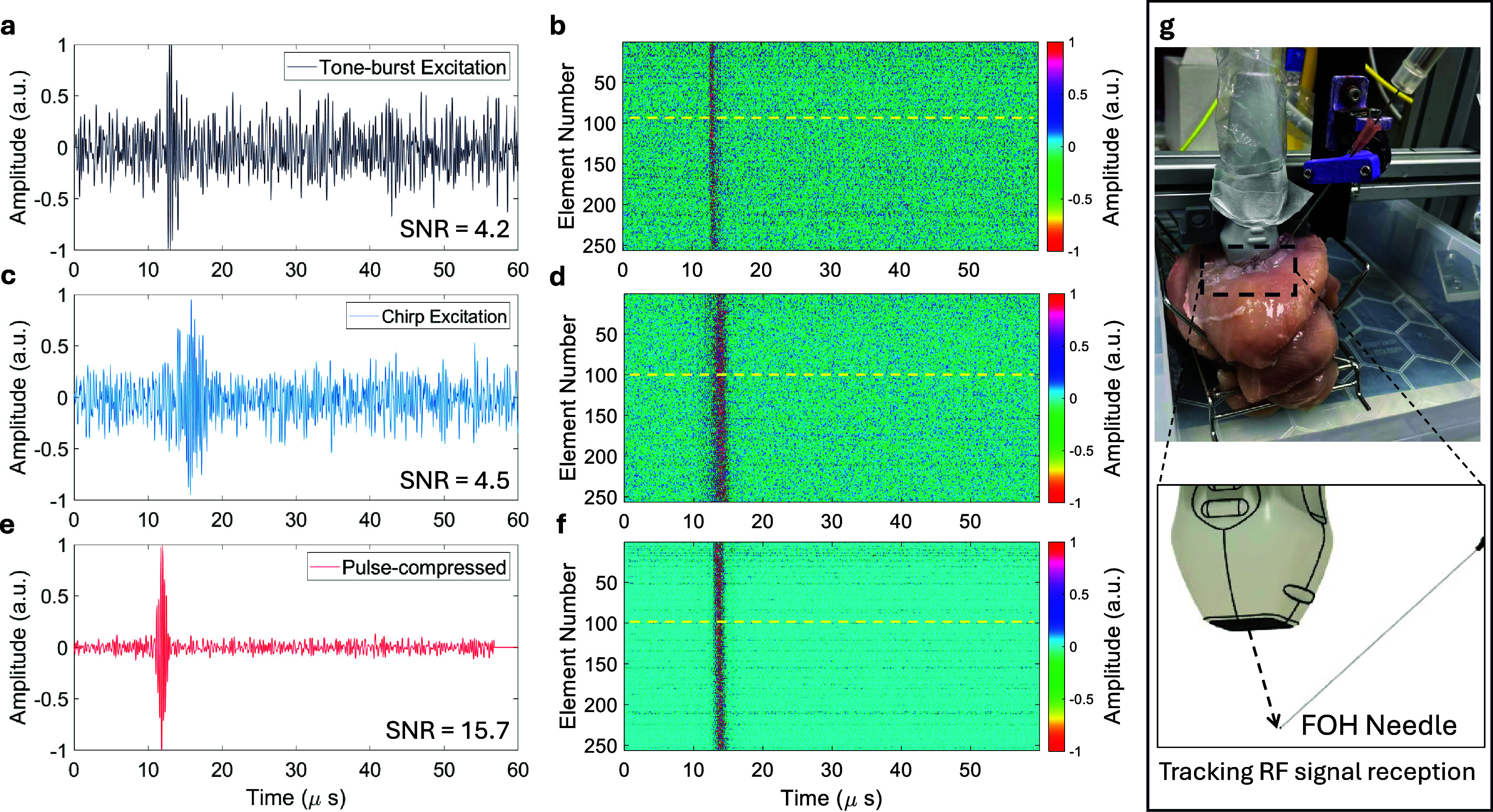
Representative examples of raw fiber-optic hydrophone (FOH) signals acquired with needle insertions into *ex vivo* chicken breast tissue. The FOH signals were recorded under three excitation conditions: (a) and (b) tone-burst excitation (four-cycle Gaussian-modulated), (c) and (d) chirped excitation (approximately 5 *µ*s duration, linear sweep from 1–5 MHz), and (e) and (f) chirped excitation after pulse compression using a time-reversed matched filter. (a), (c) and (e) show representative FOH signals with transmissions from probe element No. 100, while (b), (d) and (f) present the corresponding full sets of FOH signals from all the 256 transducer elements. (g) Photo and schematic illustration of the experimental setup.

An example of tracking RF signals acquired from the same needle site within *ex vivo* chicken breast tissue is shown in figure 7. The SNR was estimated by comparing the baseline noise level measured before 8 *µ*s with the peak amplitude of the received signal.

For tone-burst excitation (figures 7(a) and (b), channel #100), the SNR was 4.2 (linear). With chirp excitation at the same site (figures 7(c) and (d)), the SNR was 4.5, showing no significant change. However, after applying pulse compression to the chirped data (figures 7(e) and (f)), the SNR increased markedly to 15.7. Across all 256 tracking channels, SNR measurements were acquired at three randomly selected needle positions within the chicken breast ex vivo phantom, repeated over three independent insertion attempts (experimental setup shown in figure 6(g)). The mean SNR increased from $6.5 \pm 1.5$ under tone-burst excitation and $6.9 \pm 1.4$ with chirp excitation, to $15.8 \pm 0.8$ after pulse compression, corresponding to an approximately three-fold improvement relative to tone-burst excitation. A one-way analysis of variance (ANOVA) comparing tone-burst and chirp excitation revealed a modest yet statistically significant difference in SNR ($F(1, 250) = 4.79$, $p = 2.96 \times 10^{-2}$, partial $\eta^2 = 1.88 \times 10^{-2}$), corresponding to a small effect size. This result indicates that chirp excitation alone provides only a marginal improvement in SNR relative to tone-burst. In contrast, with pulse-compression, a one-way ANOVA across all three excitation strategies demonstrated a highly significant overall effect on SNR ($F(2, 250) = 742.3$, $p < 1 \times 10^{-16}$, partial $\eta^2 = 0.74$), reflecting a large effect size. Post-hoc pairwise comparisons further confirmed that pulse-compressed chirp excitation yielded significantly higher SNR than both tone-burst and uncompressed chirp excitation. Collectively, these findings indicate that the substantial SNR enhancement arises primarily from pulse compression rather than from chirp excitation alone.

### 3.4. Phantom study

**Figure 8.**
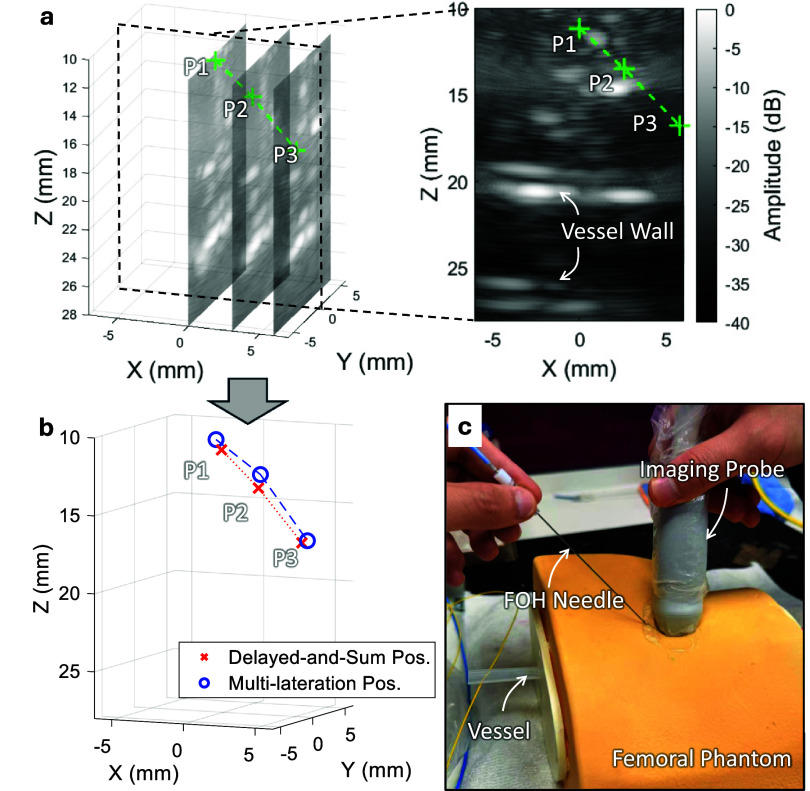
Illustration of volumetric US imaging and tracking (using a 13-element sparsified subset) during needle insertion into a femoral nerve block phantom. (a) US volume overlaid with the multilateration-based tracked needle-tip position in the imaging coordinate system; (b) comparison of 3D tracked trajectories from the same insertion attempt; (c) schematic of the experimental setup used during the procedure.

Figure 8(a) presents the volumetric US images acquired during manual needle insertion into a femoral nerve block phantom. A water-filled plastic tube, sealed at both ends, was embedded to mimic the femoral vessel, which was clearly visualized within the reconstructed volume. The overlaid needle trajectory demonstrated the system’s ability to provide real-time anatomical context alongside needle tracking.

As shown in figure 8(b), these positions were localized using both the DAS method (full 256-element array) and the multilateration method with a sparsified 13-element subset. The tracked positions from both methods aligned closely with the needle trajectory visualized in the volumetric images, confirming that the system can perform simultaneous 3D tracking and volumetric imaging in the same coordinate space. These validate the system’s capability to maintain clinically relevant tracking accuracy while preserving anatomical visualization, even with a sparsified subset of active tracking elements.

## 4. Discussion

Real-time spatial tracking of the needle tip alongside US images can significantly enhance procedural safety in many clinical applications, including regional anesthesia and pain management, solid organ biopsy and fetal surgery. This study demonstrates the feasibility of high-speed 3D tracking and imaging by integrating multilateration-based needle-tip tracking with volumetric US imaging using a needle-integrated FOH and a sparsified 2D matrix array. While high-quality B-mode imaging remains essential for anatomical visualization, the proposed approach is intended to complement rather than replace B-mode imaging. Because the FOH signals received during imaging transmissions are not suitable for tracking, as these transmissions involve multiple elements and therefore have limited angular coverage, separate imaging and tracking phases are required. Consequently, this approach does not reduce the number of transducer elements or simplify the hardware configuration. Key findings are: (1) chirp excitation with pulse compression improved SNR by approximately 3× over tone-burst excitation, enabling more reliable ToA estimation; (2) multilateration maintained an accuracy better than 2 mm with only 13 outmost active elements, achieving a theoretical acquisition rate of 45.45 Hz; and (3) accurate needle tracking was validated in water-tank experiments, and simultaneous 3D US imaging and needle tracking was further demonstrated for feasibility in ex vivo chicken breast tissue and femoral nerve block phantom studies.

The FOH offers several advantages over conventional piezoelectric transducers. FOHs are inherently broadband, enabling high–temporal-resolution ToA estimation without the bandwidth limitations of piezoelectric elements. In addition, their near-omnidirectional sensitivity supports robust needle tracking over a wide range of insertion angles (Guggenheim *et al* 2017). As demonstrated in our previous study on 2D needle tracking with a needle-integrated FOH (Xia *et al* 2015), although the SNR of the FOH signal was observed to decrease at steep insertion angles, reliable needle-tip localization was consistently achieved over clinically relevant insertion angles ranging from 15$\,^\circ$ to 61$\,^\circ$ relative to the imaging probe. These results indicate the functional robustness and reliability of FOH-based tracking across typical clinical needle insertion geometries. Their miniature size and optical nature eliminate the need for electrical cabling to the needle, reducing EM interference and improving safety in electrically sensitive environments such as MRI. In addition, FOHs are immune to electrical cross-talk and can be easily integrated into slender interventional tools without significantly altering their mechanical properties.

Compared with DAS beamforming, the multilateration approach demonstrated greater robustness under channel sparsification. While the DAS method achieved an overall tracking accuracy of $0.30 \pm 0.21\,\mathrm{mm}$ using full 256-element matrix array excitation in a water-tank experiment with a translational stage (Liang *et al* 2026), its reconstruction quality degraded rapidly as the number of active elements decreased, leading to degraded tracking accuracy (see figure 7(a)). In contrast, the multilateration method preserved stable localization performance ($1.27 \pm 0.65\,\mathrm{mm}$) under sparsified configurations by directly estimating the needle-tip position from ToA measurements, without requiring volumetric image reconstruction. Chirp excitation further enhanced multilateration by improving timing precision, which is critical given the sensitivity of range-based localization to timing jitter. Channel sparsification not only reduced data acquisition time but also increased the achievable frame rate, addressing a major limitation for real-time guidance. In addition, the tracking frame rate could be further increased by employing orthogonal coded excitations across multiple transducer elements to enable simultaneous transmission within a single trigger event, an approach that has been shown to preserve signal separability and acquisition efficiency in US systems (Yang and Chakrabarti 2012). The post-processing time of the DAS scheme on the current workstation (GPU: Quadro RTX 5000) was approximately $22.8 \pm 3.9\,\mathrm{ms}$ per frame using full 256-channel data. In contrast, the multilateration-based method required less than $0.03\,\mathrm{ms}$ per frame, representing a substantial reduction in computational demand and enabling significantly improved processing efficiency under the same hardware configuration.

With tracking accuracy assessment in water, the observed anisotropic performance, particularly the faster degradation along the *Y*-dimension, likely arises from geometric and experimental factors, including the orientation of the needle insertion relative to the US probe and the position of the needle stylet relative to the embedded FOH. These factors can introduce direction-dependent variations in signal amplitude and ToA estimation. Our tracking algorithm relies on the assumption of a homogeneous sound-speed distribution, which may introduce acoustic aberration errors in acoustically heterogeneous tissue. However, such heterogeneity similarly affects conventional US image formation, as both tracking and imaging reconstruction algorithms rely on the same assumption of a uniform sound-speed distribution. Consequently, aberration-induced errors are expected to impact both the tracked needle position and the US image in a comparable manner when imaging is used as the guidance modality to visualize patient anatomy. In this context, any systematic bias caused by sound-speed heterogeneity would likely manifest consistently in both the displayed anatomy and the tracked needle tip position, thereby preserving their relative spatial relationship. Nevertheless, we acknowledge that severe acoustic heterogeneity could introduce localisation bias and warrants further investigation. In future studies, x-ray computed tomography could be employed to provide an independent ground-truth needle position relative to the US probe, enabling quantitative assessment of the impact of sound-speed heterogeneity on tracking accuracy.

The measured tracking accuracy in water ($1.27 \pm 0.65$ mm using a sparsified subset of 13 outer elements) should be interpreted in a clinical context. For ultrasound-guided regional anaesthesia and similar needle-based interventions, commonly used needles have outer diameters on the order of 1 mm, ranging from approximately 0.71 mm for 22-gauge needles to 1.83 mm for 14-gauge needles. As such, the reported tracking accuracy is comparable to the diameters of needles used in these procedures, and is likely to be sufficient for many US-guided needle procedures. Further improvements in tracking accuracy may be achieved by incorporating advanced deep-learning-based approaches or by adopting maximum-likelihood estimation strategies, as demonstrated in our recent work (Baker *et al* 2025).

Several limitations should be acknowledged. From a hardware perspective, the current tracking performance is constrained by the limited angular coverage of the 2D matrix probe. When the needle tip is positioned with a large lateral or elevational offset from the probe’s central axis, the tracking accuracy is reduced. This limitation could be mitigated by employing a US probe with a larger footprint, or multiple US probes that are coherently operated (Peralta *et al* 2023). Additionally, the performance of the FOH can be degraded by excessive fiber bending and temperature variations. An alternative future approach could involve replacing the FOH with a fiber-optic transmitter, as proposed in Baker *et al* (2024), driven by a pulsed laser for tracking, while the sparsified subset array functions as the receiver. This configuration could improve tracking robustness, reduce overall system cost. Additionally, all the tracking data in this study were processed offline using a custom MATLAB script. For clinical translation, a fully integrated system will be required to enable simultaneous imaging and tracking with minimal processing latency. Such real-time operation could be achieved by implementing the algorithm on GPU- or FPGA-based platforms to meet the stringent speed requirements of intraoperative guidance.

## 5. Conclusion

This work is the first to demonstrate the feasibility of combining multilateration-based needle-tip tracking with volumetric US imaging using an FOH-integrated needle and a sparse 2D matrix array. Chirp excitation with pulse compression triples the tracking SNR relative to tone-burst excitation, improving ToA estimation. With a Fermat’s spiral sparsification strategy, multilateration achieved a mean accuracy of $1.27 \pm 0.65$ mm using only 13 active elements, enabling a theoretical acquisition rate of 45.45 Hz. These results underscore the system’s potential for real-time guidance of various minimally invasive procedures by simultaneous 3D anatomical visualization and accurate needle tip localization.

## Data Availability

The data that support the findings of this study will be openly available following an embargo at the following URL/DOI: https://github.com/Weidongleon/PMB_multilateration. Data will be available from 1 December 2026.
